# Association between Cardiac Auscultation and Echocardiographic Findings in Warmblood Horses

**DOI:** 10.3390/ani11123463

**Published:** 2021-12-05

**Authors:** Jakob Hövener, Julie Pokar, Roswitha Merle, Heidrun Gehlen

**Affiliations:** 1 Equine Clinic Bargteheide, 22941 Bargteheide, Germany; J.Pokar@pferdeklinik-bargteheide.de; 2 Institute for Veterinary Epidemiology and Biostatistics, Freie Universitaet Berlin, 14163 Berlin, Germany; Roswitha.Merle@fu-berlin.de; 3 Equine Clinic, Veterinary Department, Freie Universitaet Berlin, 14163 Berlin, Germany

**Keywords:** cardiology, equine, auscultation, echocardiography, heart murmur, valvular regurgitation

## Abstract

**Simple Summary:**

In our study, we retrospectively analyzed cardiac examinations in a large number of warmblood horses conducted over a period of almost 20 years. We compared the results of the cardiac auscultation as the character and grade of a heart murmur with the results of the echocardiographic examination. We found that auscultation works very well to identify the valve affected, if following the general clinical guidelines on which kind of murmur is usually caused by specific valvular regurgitations. Auscultation is less specific in determining the grade of the regurgitation based on the loudness of the murmur. Only low-grade murmurs are usually caused by mild regurgitations, while differentiation between moderate and severe regurgitations based on the loudness of the murmur is not reliable. Moreover, we could not find that enlargement of one or more compartments of the heart generally leads to a higher-grade murmur.

**Abstract:**

Heart murmurs are detected frequently when auscultating horses and certain murmurs can usually be linked to specific valvular regurgitations. Limited information exists about the accuracy of these broad rules in warmblood horses and the influence of grade of the regurgitation and dimensional changes on murmur intensity. This study aims to clarify the accuracy of cardiac auscultation in warmblood horses and the influence of the grade of regurgitation and dimensional changes on the loudness of the murmur. In this retrospective study, 822 warmblood horses presented for cardiac examination in a large equine referral center in northern Germany underwent a thorough cardiac auscultation. In total, 653 of these revealed one or more heart murmurs. Most common auscultatory findings were left-sided systolic murmurs (68%) or left-sided diastolic murmurs (15%). On 635 of these horses, an echocardiographic examination was performed, revealing regurgitations of the mitral valve as the most common valvular regurgitation (77%) followed by regurgitations of the aortic valve (23%). Thirty-one percent of horses that underwent echocardiographic examination displayed dimensional changes of one or more compartments of the heart, with the left atrium being most affected (21%), followed by the left ventricle (13%). The main goal of this study was to link certain auscultatory findings with results of the echocardiographic examinations, trying to determine whether auscultation and echocardiography agreed on the valve affected, as well as to find out if loudness of the murmur coincided with grade of regurgitation and presence of dimensional changes. Agreement between auscultation and cardiac ultrasound was substantial (Kappa 0.74) if one or more murmurs and regurgitations were present and almost perfect (Kappa 0.94) if only one murmur and one regurgitation were found. Auscultation was particularly well suited for detection of left-sided systolic and diastolic murmurs, with 87% of left-sided systolic murmurs being caused by a mitral valve regurgitation and 81% of left-sided diastolic murmurs originating from an aortic valve regurgitation. We found a fair agreement between the grade of regurgitation and the respective murmur. Association was particularly good between mild regurgitations and low-grade murmurs, while differentiation between moderate to severe regurgitation based upon the loudness of the murmur was less reliable. Dimensional changes were usually linked to more severe regurgitations and higher-grade murmurs. However, a direct correlation between murmur intensity and the presence or severity of dimensional changes, independent of the grade of valvular regurgitation, could not be established in this cohort of horses.

## 1. Introduction

Heart murmurs are a common auscultatory finding in horses of all breeds, ages and disciplines and can be of a physiological or pathological nature [1,2]. While physiological murmurs result from the large amount of blood and the high velocity of the blood flow in the equine cardiovascular system, whose enormous size presents ideal conditions for the formation of turbulent blood flow, pathological murmurs result from either stenosis or regurgitation of one of the valves of the heart [2]. Stenosis is rarely seen in horses and most pathological systolic or diastolic murmurs are caused by valvular regurgitations or congenital defects [1,2]. This may make linking auscultatory and echocardiographic findings easier than, for example, in dogs, cats or humans, who are commonly affected by valvular stenosis, as the list of common differential diagnoses is shorter [3,4,5]. However, evaluating the significance of cardiac auscultatory findings still poses a major challenge, as it can be difficult to assess what cardiac disease is underlying these murmurs, how severe it is and what effect it may have on the performance, safety and life expectancy of the horse [1,6]. Further diagnostics are usually needed to assess the kind and severity of the underlying cardiac condition and decide whether a potential regurgitation could affect a horse’s short- and long-term performance [7]. However, despite the multitude of modern diagnostic tools available in equine referral centers today, thorough auscultation of the heart is still one of the most important and sometimes the only available diagnostic tool to evaluate potential heart disease in equine veterinary practice [8,9,10]. Linking certain auscultatory findings with specific valvular regurgitations has been established—for example, a left-sided systolic murmur with a regurgitation of the mitral valve [2]—thus, detailed auscultation is supposed to enable the experienced veterinarian to make a list of differential diagnoses or even pinpoint a specific diagnosis based on the findings [7]. However, it is widely discussed whether auscultation of a higher-grade murmur indicates a more severe valvular regurgitation or may even be due to dimensional changes of the heart [7,8]. This paper aims to describe the auscultatory and echocardiographic findings in a large group of warmblood horses and evaluate to what extent auscultatory findings concur with the results of echocardiography and whether there is an association between the grade of a murmur and that of a valvular regurgitation or even the presence or grade of dimensional changes.

## 2. Material and Methods

Horses—Warmblood horses that were presented for initial cardiac work-up in a large equine referral center in northern Germany between January 2000 and May 2020 were included in the study. Results from follow-up examinations were not included; therefore, each horse appears only once in the study. The horses were presented because of either a clinical complaint such as poor performance or collapse or because of a heart murmur or arrhythmia detected as an accidental finding. A detailed report of the examination was saved in the patient file for all horses, including information about the clinical examination, the auscultatory findings and, if conducted, the results of the echocardiography.

Auscultation—After a general clinical examination with a special focus on the cardiovascular system, the horses presented were auscultated by one of five experienced veterinary practitioners upon arrival in the clinic. Auscultation was performed in a quiet environment on the left and right hemithorax with a conventional stethoscope placed in the third, fourth and fifth intercostal space. Frequency, intensity and rhythm of the heartbeats, and audible murmurs were noted. All auscultated heart murmurs were described as audible loudest on the left, right or on both sides of the thorax. Moreover, they were characterized depending on their timing as systolic, diastolic or continuous murmurs and were graded from Grade 1 to Grade 6 (Table 1) [2].

Echocardiography—An echocardiographic examination was performed if a murmur was present upon auscultation. This included an examination in B-Mode, M-Mode and a color Doppler-echocardiography of all four valves [8,11]. If regurgitations could be detected, they were graded as mild, medium or severe, depending on the size of the jet [8,12]. If any dimensional changes were present, the maximal size of the atrium or chamber was measured in millimeters and recorded [11]. The LA and LV of adult warmblood horses were considered enlarged if the compartment measured more than 135 mm. Measurements larger than 80 mm for the RA and RV were considered enlarged [8,13]. Dimensions were judged relative to their size for foals and subadult horses. Any other abnormal findings were recorded in the patient file as well. If documentation of the echocardiographic exam was insufficient, the images and videos taken during the examination were reviewed from the video archive and were reevaluated by an experienced clinician.

Data Analysis—The auscultatory and echocardiographic findings were collected retrospectively from the reports saved in the electronic patient files and the video archive. Horses were classified in different groups for the statistical analysis. Criteria were the presence of a murmur at the time of the examination, grade, phase and point of maximal intensity of that murmur, and the findings of the echocardiographic examination, such as the presence and grade of valvular regurgitation and dimensional changes.

Data were stored in an Excel sheet (IBM Excel^®^ Version 2016, Microsoft Corporation, Redmont, WA, USA) and analyzed using IBM SPSS statistical software version 25 (SPSS Inc., Chicago, IL, USA). Descriptive statistics are presented in terms of frequency tables as well as means, median, standard deviations, 95% confidence intervals and boxplots.

The main aim of the statistical analysis was to determine the accuracy of auscultation in identifying the valve affected, and grading of valvular regurgitations. Moreover, we investigated whether the presence of dimensional changes of the heart altered murmur intensity. Whether associations exist between the presence and severity of valvular regurgitations and the presence of dimensional changes to be better able to proportion the association between auscultation and echocardiography was also evaluated.

In order to determine the accuracy of auscultation to diagnose valvular regurgitations, we used points of maximum intensity and the phase in which the murmur was audible and then linked this with the most probable valvular regurgitation. As described by Bonagura and Reef [14], a left-sided systolic murmur was linked to MVI; left-sided diastolic murmur to AVI; and right-sided systolic murmur was allocated to TVI or a VSD.

The six grades of murmurs were categorized into 3 degrees of severity to allow comparisons with the severity of valvular regurgitation. Murmurs of Grades 1 and 2 were classified as low-grade, murmurs of Grades 3 and 4 as moderate, and murmurs of Grade 5 and 6 as high-grade murmurs.

A Cohen’s Kappa test was used to determine agreement between auscultatory and echocardiographic findings. According to McHugh [15], the Kappa coefficient was assessed as follows: 0 = poor; >0–0.2 = slight; >0.2–0.4 = fair; >0.4–0.6 = moderate; >0.6–0.8 = substantial; >0.8–1.0 = almost perfect.

A Kolmogorov–Smirnov and a Shapiro–Wilk test were used to determine whether the severity of the valvular regurgitation and the grade and presence of dimensional changes were normally distributed for different murmur intensities. Since data were not normally distributed, a Mann–Whitney U test was used to determine whether murmur intensity differed between horses with and without dimensional changes of one or more compartments of the heart.

Furthermore, odds ratios (OR) were calculated to determine whether specific cardiac findings lead to an increased risk of the development of other cardiological pathologies. Risk is enhanced if the OR is above 1.00.

An analysis of variances (ANOVA) was carried out to test how the different grades of regurgitation and the different grades of dimensional changes influenced the murmur intensity. The target variable was the loudness of the murmur. Input variables were the different grades (no, mild, moderate, severe) of MVI, AVI and TVI, the different grades (no/<135 mm, mild/135–140 mm, moderate/141–150 mm, severe/>151 mm) of left atrial and left ventricular dimensions, and the different grades (no/<80 mm, mild/81–90 mm, severe/>91 mm) of right atrial and right ventricular dimensions. All two-way interactions were also included in the model. The homogeneity of the different variances was determined using Levene’s test. Equal variances could be assumed, as *p* was above 0.05 (*p* = 0.543). Hochberg’s post hoc test was then used for multiple comparisons. Model diagnostics included a visual inspection of normality and homoscedasticity of residuals. The level of significance was set at 0.05.

## 3. Results

Horses—822 warmblood horses were included in the study. They were between 3.5 months and 29.6 years of age. The mean age was 9.17 years, while the median age was 7.67 years. Most horses were Hanoverians (*n* = 264), followed by Holsteins (*n* = 229), Trakehners (*n* = 98), Oldenburgs (*n* = 85) and German Riding Horses (*n* = 78). A total of 242 (29%) of the horses were mares, 72 (9%) were stallions and 509 (62%) were geldings.

Auscultation—Auscultation was performed in all 822 horses with findings in 721 of the horses. One or more murmurs could be auscultated in 553 horses and a murmur combined with an arrhythmia in 100 horses. Only an arrhythmia could be auscultated in 68 horses. A total of 81% of all murmurs were systolic (*n* = 528) with a left-sided systolic murmur being the most common with a total of 445 cases (68%). A right-sided systolic murmur was present in 46 cases (7%) and the systolic murmur was of a bilateral nature in 37 cases (5.5%). Diastolic murmurs were less frequent findings with a total of 109 cases (17%). A total of 95 (15% of all murmurs) of the diastolic murmurs were left-sided, 1 (0.2%) was right-sided and 13 (2%) both-sided (Table 2).

Echocardiography—An echocardiographic examination was performed in 735 cases and revealed pathologies in 632 cases. One or more regurgitations were present in 601 of the 735 horses (82%) examined. The most common finding was an isolated MVI, which was found in 372 horses (45%), followed by an isolated AVI, which was present in 87 horses (11%). Further common findings were a combined MVI and TVI (*n* = 47/6%), a combined MVI and AVI (*n* = 29/4%) and an isolated TVI (*n* = 25/3.5%) (Table 2). An MVI was present in a total of 467 horses, either isolated or combined with other findings. An AVI was present in 138 horses, TVI in 88 horses and PVI in 5 horses. Most regurgitations were graded as mild; however, AVI were more often graded as moderate or severe than MVI, TVI or PVI (Table 3). A VSD was found in 20 horses.

No underlying regurgitation could be detected in 46 cases (7%) of all cases, even though a murmur was present upon auscultation. These murmurs were left-sided systolic in 23 cases, left-sided diastolic in 12 cases and right-sided systolic in 6 cases.

Dimensional changes of one or more departments of the heart were present in 228 (31%) of the 735 horses examined echocardiographically. Left atrial enlargement was the most common finding, being present in 154 horses (21%), followed by left ventricular enlargement found in 95 (13%) of the horses examined. RA enlargement was present in 21 (3%) of the horses and 19 horses (2%) were diagnosed with dimensional changes of the RV.

Association between different echocardiographic findings—Dimensional changes and valvular insufficiencies were a combined finding in 200 cases. This means that 88% of all horses with dimensional changes also had one or more valvular regurgitations and 34% of all horses with valvular regurgitation could also be diagnosed with dimensional changes of one or more compartments of the heart.

The risk for horses with valvular regurgitations to also have dimensional changes was more than twice as high as for horses without regurgitations (OR = 2.12). Moreover, a higher-grade regurgitation leads to a higher risk of dimensional changes, with the risk being six times higher in moderate to severe MVI (OR = 5.92) and seven times higher in moderate to severe AVI (OR = 6.72) than in mild regurgitation of these valves.

Association between auscultatory and echocardiographic findings—Only one murmur and one regurgitation were present in each of the 458 horses. Kappa revealed an almost perfect agreement (Kappa 0.94, *n* = 458) between auscultation and echocardiography in these horses. If horses with more than one murmur and more than one regurgitation were taken into consideration (*n* = 547), agreement was slightly less but still substantial (Kappa 0.74, *n* = 547). A total of 430 of 447 (87%) left-sided systolic murmurs were due to an MVI. This means, in return, that 96% of all MVIs were accompanied by a left-sided systolic murmur. Therefore, agreement between auscultation and echocardiography was substantial (Kappa 0.68, *n* = 653) in this case. A total of 99 of 123 (81%) left-sided diastolic murmurs were linked to an AVI, ergo 74% of all AVIs presented as left-sided diastolic murmurs on auscultation. There is substantial agreement (Kappa 0.72, *n* = 653) between these two diagnostic tests. Right-sided systolic murmurs were due to a TVI in 22 (48%) and/or due to VSD in 13 (29%) out of 46 cases. This means a murmur of that kind was due to one of these two conditions in 76% of cases. Agreement (Kappa 0.51, *n* = 653) was moderate in this case. However, while 65% (*n* = 13) of all VSDs were right-sided systolic murmurs on auscultation, only 26% (*n* = 22) of TVIs presented themselves as right-sided systolic murmurs (Table 2).

**Figure 1:**
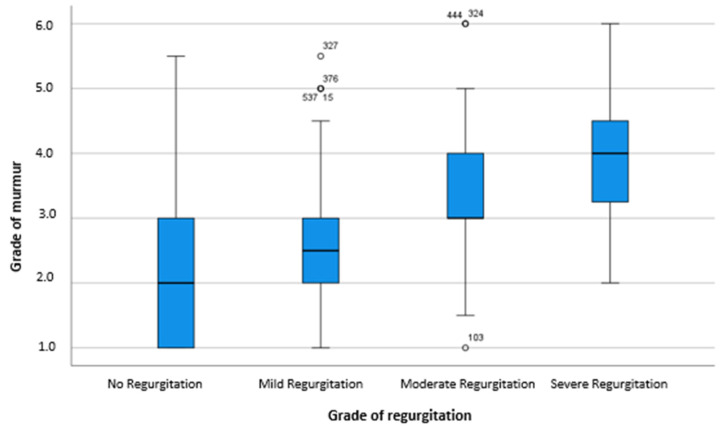
Association between grade of murmur and severity of regurgitation.

We detected a fair agreement (Kappa 0.32, *n* = 562) between the grade of the murmur and the severity of the valvular regurgitation. Agreement was particularly good for low-grade murmurs, meaning that 236 of 276 (86%) low-grade murmurs also presented as mild regurgitations in echocardiography. Only 13% (*n* = 37) of these low-grade murmurs were linked to moderate regurgitations and only 1% (*n* = 3) to a severe regurgitation. Agreement was less for moderate murmurs. Of the 257 moderate murmurs detected on auscultation, only 40% (*n* = 102) could be linked to a moderate regurgitation in the ultrasonographic examination of the heart, while 48% (*n* = 122) of moderate murmurs were related to a mild and 22% (*n* = 33) to a severe regurgitation. Concerning high-grade murmurs, 13 of 29 (45%) could be linked to a moderate and only 11 (40%) to a severe regurgitation. However, only 5 (17%) high-grade murmurs were associated with a mild insufficiency (Table 4, Figure 1).

**Figure 2:**
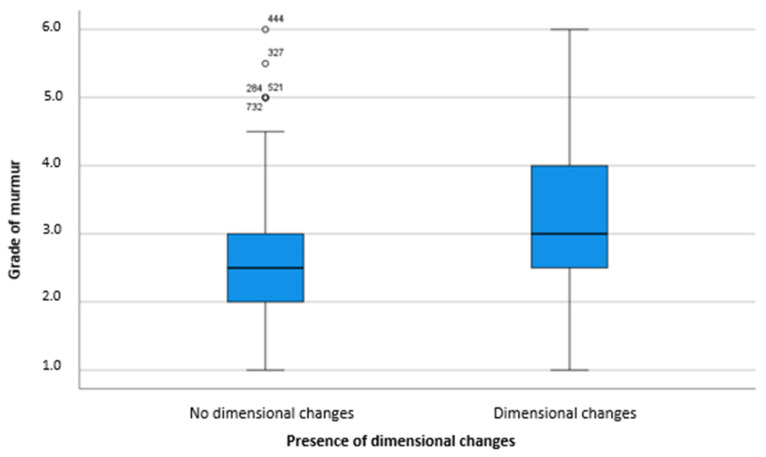
Difference in murmur intensity between horses with and without dimensional changes of the heart.

Horses with an enlargement of one of the compartments of the heart most frequently had a murmur of a moderate grade (*n* = 117/51%) upon auscultation. On the other hand, the most frequent finding in horses without dimensional changes was a low-grade murmur (*n* = 278/47%) (Table 5). The mean murmur intensity in horses with dimensional changes of one or more compartments of the heart was significantly louder (3.3 ± 1.15) than in horses without any dimensional changes (2.5 ± 0.1) (Figure 2). This finding was supported by a Mann–Whitney U test, which revealed that significant differences exist regarding the murmur intensity between horses with and without dimensional changes of one or more compartments of the heart (U = 64911.5, *p* < 0.001). ANOVA showed that grades of MVI, TVI, AVI, and VSD as well as dimensional changes of the LA and of the RA and RV were significantly associated with murmur intensity (Table 6). While TVI (*p* < 0.001), VSD (*p* < 0.001), and dimensional changes of the RA (*p* = 0.010) influenced the murmur intensity directly, the other factors had significant interactions: AVI interacted significantly with MVI (*p* = 0.009) and with TVI (*p* = 0.010). Changes in the dimension of the LA showed significant interactions with dimensional changes of the RA (*p* = 0.016), with MVI (*p* = 0.001) and with VSD (*p* = 0.015). The MVI also interacted with the dimensional changes of the left ventricle (*p* = 0.008).

Following Hochberg’s post hoc test, all categories of MVI differed significantly from each other (*p* < 0.05), with no and mild MVI causing systolic murmurs of lower intensities than moderate and severe MVI. This test revealed for TVI that only severe regurgitation differed significantly in loudness from the other grades of regurgitation (*p* < 0.001). No significant difference could be detected between mild and moderate regurgitation (*p* = 0.963). No and mild insufficiencies differed significantly from moderate and high-grade regurgitations in loudness in regurgitations of the aortic valve according to Hochberg’s post hoc test (*p* < 0.001). Moderate insufficiencies were considerably louder than mild regurgitations (*p* < 0.001) and quieter than severe regurgitations (*p* = 0.006), which were markedly louder than mild and moderate regurgitations (*p* < 0.001 and 0.006, respectively). Concerning the LA, hearts without dimensional changes (<135 mm) differed significantly from all other groups (*p* ≤ 0.001). The larger the LA, the higher the murmur intensity. Dimensional changes of the LV always led to significant changes of the murmur (*p* < 0.001). However, there was no significant difference between mild (136–140 mm) and moderate (141–150 cm) changes (*p* = 0.951), with the murmur intensity being lower the larger the ventricle. The categories of the dimensional changes of the RA did not differ significantly, except that the group of no changes (<80 mm) had a significantly higher murmur intensity than the group of severe (>91 mm) changes (*p* = 0.013). The same situation applied to the RV: only no (<80 mm) changes were associated with a lower murmur intensity than severe (>91 mm) changes (*p* = 0.031).

**Figure 3:**
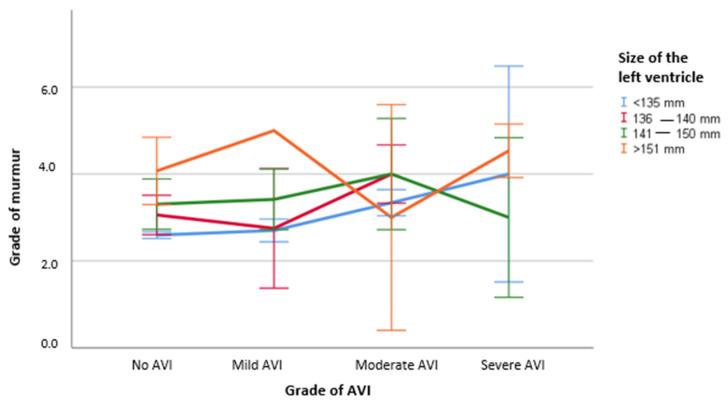
Association between intensity of left-sided diastolic murmurs, grades of AVI and grades of left ventricular enlargement. Abbreviations: AVI = Aortic Valve Regurgitation.

Finally, in Figure 3, the relationship between the intensity of left-sided diastolic murmurs, grade of AVI and dimensional changes of the LV is displayed in Figure 3. It becomes clear that the murmur intensity increased with the grade of the AVI when no dimensional changes of the LV were present and when LV enlargement was mild (136–140 mm). However, when the enlargement was moderate (141–150 mm), mean murmur intensity was 3.5/6 in mild regurgitations, 4 in moderate regurgitations and only 3 when severe regurgitation was present. If a severe left ventricular enlargement was present (>141 mm), mild AVI was combined with a mean murmur intensity of grade 5, moderate AVI with a mean murmur intensity of grade 3 and severe AVI with a mean murmur intensity of grades 4 and 5.

## 4. Discussion

Equine veterinarians are frequently asked to evaluate heart murmurs upon special request, due to clinical concerns such as poor performance or an accidental finding on a prepurchase or a routine examination. As severe cardiac disease can be a safety risk for the horse and rider, it is crucial to find out the etiology of auscultatory findings and, consequently, judge the horse’s prognosis [16]. Moreover, valvular regurgitations are described as a cause of poor performance in horses [17] and detection of a murmur during a prepurchase examination can prevent the sale of an otherwise healthy horse [18]. Verdegaal et al. [19], for example, found that 36% of 77 warmblood horses diagnosed with a murmur during a prepurchase examination were not sold and 21% were sold for a price lower than originally assessed. This was despite the fact that the horses did not show any clinical signs of heart disease. A correct classification of a murmur and the underlying heart disease is of utmost importance due to these important economic concerns for the equine industry and the potential safety hazard. Consequently, thorough cardiac auscultation is a cheap and normally available diagnostic tool in equine practice and can, if performed correctly, provide an insight into what pathology is causing the murmur and the significance it may have. Therefore, it is usually regarded as a fairly specific tool [7,14,20].

However, the accuracy of auscultation is widely discussed in the literature and different studies have come to very different conclusions regarding the significance and exactness of auscultatory findings [21]. Naylor et al. [22], for example, described the accuracy in detecting the cardiac pathology underlying the murmur as highly dependable on the training of the examiner. They found that internal medicine specialists came to the right diagnosis based on auscultation alone in 53% of all cases. General equine practitioners, on the other hand, came to the correct conclusion in 33% and veterinary students in 29% of all cases [22]. In other studies, the specificity of auscultation to diagnose atrioventricular regurgitations correctly was described as being up to 100% [23]. In the same study, the positive predictive value of detecting MVI and TVI in thoroughbred racehorses was described as being 100% as well [23]. Other studies also found a very significant association between murmurs over the mitral, aortic or tricuspid area and regurgitations detectable in Color-Doppler Echocardiography of the respecting valve [24,25]. In one study on standardbred racehorses, 55% of tricuspid valve insufficiencies were accompanied by a respective audible murmur, while this was only the case in 15% of MVI and 9% of AVI [26]. Auscultation and echocardiography also agreed on the valve affected in most cases in a study on a population of mainly warmblood horses [19]. In our study of warmblood horses examined auscultatorily and echocardiographically, we found that agreement between murmur and regurgitation was 94% if only one murmur and one regurgitation were present. Agreement between auscultation and echocardiography was 87% for MVI and 81% for AVI, while only 26% for TVI and 37% for VSD. This is probably due to the fact that right-sided systolic murmurs always have two differential diagnoses, being TVI and VSD [14]. Considering this, the presence of a right-sided systolic murmur concurred with either a TVI or a VSD in 76% of cases. Agreement was slightly worse when cases with more than one murmur and/or more than one regurgitation were considered. However, agreement between auscultation and echocardiography was still 76% in these cases. This makes the auscultatory accuracy of the certified equine practitioners conducting the examination in this case significantly better than described in other studies, in which accuracy is labeled with only 33% [22] or even less [26]. A 100% accuracy, as described by Young and Wood [23], could not be achieved. However, this very high accordance was achieved examining young thoroughbred racehorses and, as Kriz et al. [25] stated, the average thoroughbred is of a much lighter build and usually has a thinner coat than a warmblood horse. Therefore, auscultation may be easier and, thus, more reliable in these smaller, delicate horses than in a larger, muscular riding horse [25]. Accordance could be even higher if character were taken into account. This makes the differentiation between functional and pathological murmurs even more reliable than if only timing and location are used as references [2]. Moreover, if right-sided systolic murmurs are present, it is easier to differentiate between TVI and VSD if the murmur is further characterized [2]. Unfortunately, due to the retrospective nature of the study, this information was not available in many cases and, therefore, could not be used for the further characterization of murmurs. This certainly presents a limitation regarding the results of this study. However, auscultation comes to the right conclusion in a lot of cases, even though the murmurs are not further characterized in this study. Therefore, assessing the location and timing of a murmur can give a good indication of the underling pathology. When examining a horse, one should, nevertheless, fully characterize a murmur as described in the literature [2] to be better able to differentiate between different pathologies and distinguish functional from pathological murmurs.

Studies in human medicine have shown that a significant correlation exists between the severity of regurgitations of the aortic valve and the intensity of diastolic murmurs, and between the loudness of systolic murmurs and the intensity of MVI [27]. The same seems to be true in small animal veterinary science. Caivano et al. [28] found that dogs with low-intensity murmurs very rarely suffered from severe pulmonic or subaortic stenosis. Dogs with a higher-grade heart murmur, on the other hand, were frequently diagnosed with severe stenosis and it was found that the severity of stenosis increased with murmur intensity [28]. This finding could be confirmed by other studies, who also found a significant association between murmur intensity and disease severity in over 500 dogs of various breeds [28,29]. It is possible that auscultation in horses may be less accurate due to the large size of the animal. It is described that low-grade murmurs can be caused by severe valvular regurgitation, and high-grade murmurs by mild regurgitations in horses [30]. However, Blissit and Bonagura [24] detected that louder murmurs correlated with significantly larger jets of longer duration, and it is described that louder murmurs are due to more severe valvular dysfunction [21]. It was also stated that mild murmurs less than grade 3/5 in horses without symptoms of cardiovascular disease are usually due to mild regurgitations [31]. We found that a fair agreement existed between the grade of the murmur and the severity of the regurgitation in our population. This was particularly true for low-grade murmurs, which coincided with 86% of mild valvular regurgitations. Therefore, it was very rare (1%) that a murmur of grade 1 or 2 was due to a severe regurgitation, giving the examiner some indication that the underlying heart disease may not be too grave. However, we could not find a significant difference in the mean murmur intensity between moderate and severe regurgitations, making a differentiation between these two based solely on auscultation very difficult. This is supported by the fact that a large proportion (45%) of severe regurgitations were on auscultation murmurs of grade 3 to 4 and only 40% of murmurs were of a higher grade. This may lead to a false sense of safety in these horses and underlines the importance of an echocardiographic examination in any horse with a left-sided systolic murmur louder than grade 3/6, a right-sided systolic murmur either louder than grade 4/6 or when a VSD is suspected and any left-sided diastolic murmurs, with a suspicion of AVI, as described in the latest consensus statement on equine heart disease [32].

Finally, we wondered whether a higher-grade murmur may also be an indicator of dimensional changes of one or more compartments of the heart. It has been found in dogs that animals with murmurs of a low grade did not show structural changes of the heart in 90% of cases and that the probability of congestive heart failure increased with murmur intensity [29]. The same seems to be true for cats [33]. On the other hand, very little is known about the association between these two findings in horses. Gehlen et al. [34] found that the dimensions and grade of murmurs increased from the first to the second examination in a follow-up examination of warmblood horses. However, no direct link between these findings could be detected [34]. In our study, the mean murmur intensity in horses with dimensional changes was 3.3/6 and, therefore, higher than in those without dimensional changes in which mean murmur intensity was 2.5/6. However, we also found that horses with a higher-grade regurgitation were more likely to have dimensional changes of the heart. Moreover, there is a fair agreement between the grade of the murmur and the severity of the regurgitation. Therefore, these results could be altered by the fact that dimensional changes are more common in horses with more severe regurgitations, and more severe regurgitations lead to higher grade murmurs. We performed a Hochberg test, as described above, to determine the influence of cardiac dimensions on murmur intensity. This showed that significant differences in murmur intensity exist between horses without or with mild, moderate or severe dimensional changes of the different departments of the heart. However, we cannot state that louder murmurs are generally indicative of dimensional cardiac changes. We found that changes in cardiac dimensions altered the loudness of the murmur; however, they do not always increase the murmur intensity but sometimes even decrease the volume of the audible murmurs. Enlarged left ventricular dimensions, for example, which proved to alter the murmur intensity separately from the grade of the regurgitation of the aortic valve, do not always increase the murmur intensity compared to hearts with normal dimensions. Therefore, we cannot state that a louder left-sided diastolic murmur is due to an AVI with underlying changes in cardiac dimensions, as, for example, a moderate AVI in horses without dimensional changes leads to, on average, a murmur of grade 3/6, which is also true for a moderate AVI in a horse with severe left ventricular enlargement.

## 5. Conclusions

Ultimately, we can say that cardiac auscultation is an integral part of any cardiac examination and very well suited to identify the cause of a murmur in case of left-sided isolated murmurs and MVI and AVI, which show an almost perfect agreement between auscultation and ultrasonography. If right-sided murmurs and more than one regurgitation are present, agreement is lower and the technique is less suited to identify the valvular regurgitation as being the cause of the murmur. The limiting factor in this case is that only the grade, timing and location were used to describe the murmur. If character could have been taken into account, a higher agreement may have been achieved.

We could determine a fair agreement between the grade of the murmur and the severity of the regurgitation. This is particularly true for mild regurgitations. Accuracy is lower for moderate- to high-grade regurgitations, with the murmur often being less loud than the severity of the corresponding valvular regurgitation may suggest, possibly introducing the risk of underestimating the risk of a grade 3 or 4 murmur. We also found that horses with cardiac dimensional changes have, on average, louder murmurs; however, this could also be due to the fact that dimensional changes are often accompanied by more severe regurgitations. In the case of AVI and left ventricular enlargement, we can even say that there is no direct correlation between the grade of enlargement and the murmur intensity.

To sum up, we can say that auscultation is very well suited for an approximate assessment of a cardiac disease, especially in rural equine practice or when transportation to an equine referral center is not possible or not wanted. However, if further sporting activities are desired, further diagnostics should be performed, especially in a moderate to loud murmur, a right-sided murmur, or multiple murmurs, as auscultation may be inaccurate in these cases.

## Figures and Tables

**Table 1:** Heart murmur grading scheme.

Grade 1	A very quiet murmur audible with careful auscultation over a localized area
Grade 2	A quiet, but over a localized area, immediately audible murmur with a lower intensity than the first and second heart sound
Grade 3	An immediately audible murmur over a larger area, with the same intensity as the first and second heart sound
Grade 4	A loud murmur, which is audible over a large area and louder than the first and second heart sound
Grade 5	A loud murmur with a precordial thrill, only audible if the stethoscope is touching the skin surface
Grade 6	A very loud murmur which is audible without the stethoscope touching the skin surface, where the first and second heart sound are no longer audible

**Table 2:** Auscultatory and echocardiographic findings. Data from horses that underwent cardiac auscultation and echocardiography.

	MVI	AVI	TVI	VSD	MVI + AVI	MVI + TVI	MVI + VSD	AVI + TVI	Other *	Total
**Systolic left-sided M.**	345 (78%)	3 (0.7%)	4 (0.9%)	0 (0%)	17 (4%)	18 (4%)	0 (0%)	2 (0.4%)	56 (13%)	445 (100%)
**Systolic right-sided M.**	1 (2%)	0 (0%)	17 (37%)	6 (13%)	0 (0%)	5 (11%)	3 (7%)	0 (0%)	14 (30%)	46 (100%)
**Diastolic left-sided M.**	1 (1%)	68 (72%)	1 (1%)	0 (0%)	4 (4%)	0 (0%)	0 (0%)	3 (3%)	18 (19%)	95 (100%)
**Diastolic right-sided M.**	0 (0%)	0 (0%)	0 (0%)	0 (0%)	0 (0%)	0 (0%)	0 (0%)	0 (0%)	1 (100%)	1 (100%)
**Systolic both-sided M.**	7 (19%)	0 (0%)	0 (0%)	1 (3%)	0 (0%)	24 (65%)	3 (8%)	0 (0%)	2 (5%)	37 (100%)
**Diastolic both-sided M.**	0 (0%)	12 (92%)	0 (0%)	0 (0%)	1 (8%)	0 (0%)	0 (0%)	0 (0%)	0 (0%)	13 (100%)
**Continuous left-sided M.**	0 (0%)	1 (13%)	1 (13%)	0 (0%)	4 (50%)	0 (0%)	0 (0%)	0 (0%)	2 (25%)	8 (100%)
**Continuous right-sided M.**	0 (0%)	0 (0%)	0 (0%)	0 (0%)	0 (0%)	0 (0%)	0 (0%)	0 (0%)	1 (100%)	1 (100%)
**Continuous both-sided M.**	0 (0%)	0 (0%)	0 (0%)	0 (0%)	1 (14%)	0 (0%)	0 (0%)	0 (0%)	6 (86%)	7 (100%)
**No M.**	18 (72%)	3 (12%)	2 (8%)	0 (0%)	2 (8%)	0 (0%)	0 (0%)	0 (0%)	0 (0%)	25 (100%)
**Total**	372 (55%)	87 (13%)	25 (4%)	7 (1%)	29 (4%)	47 (7%)	6 (1%)	5 (1%)	100 (15%)	678 (100%)

Abbreviations: M. = Murmur, MVI = Mitral valve regurgitation, AVI = Aortic valve regurgitation, TVI = Tricuspid valve regurgitation, VSD = Ventricular septal defect. *, e.g., PVI, physiological regurgitations, myocardial and pericardial disease, complex congenital disease, neoplasia, modified motion patterns.

**Table 3:** Grade of valvular regurgitations. Data from 735 horses that underwent echocardiography.

	Mild	Moderate	Severe	Total
MVI	337 (72%)	108 (23%)	22 (5%)	467 (100%)
AVI	73 (53%)	44 (32%)	21 (15%)	138 (100%)
TVI	67 (76%)	11 (13%)	10 (12%)	88 (100%)
PVI	5 (100%)	0 (0%)	0 (0%)	5 (100%)
Total	482 (69%)	163 (23%)	53 (8%)	698 (100%)

Abbreviations: MVI = Mitral valve regurgitation, AVI = Aortic valve regurgitation, TVI = Tricuspid valve regurgitation, PVI = Pulmonal valve regurgitation.

**Table 4:** Association between the grade of murmur and the severity of valvular regurgitation. The loudest audible murmur and the most severe regurgitation were compared if multiple murmurs or regurgitations were present. The severity of regurgitation was based on Color Doppler Echocardiography.

Murmur Intensity:	No Regurgitation	Mild Regurgitation	Moderate Regurgitation	Severe Regurgitation
No Murmur	90 (78%)	22 (19%)	3 (3%)	0 (0%)
Grade 1–2/6	37 (12%)	236 (75%)	37 (12%)	3 (1%)
Grade 3–4/6	18 (7%)	122 (44%)	102 (37%)	33 (12%)
Grade 5–6/6	3 (9%)	5 (16%)	13 (41%)	11 (34%)
Total	148	363	152	47
95% Confidence Interval				
Lower Bound	1.984	2.428	3.131	3.710
Upper Bound	2.637	2.578	3.412	4.269

**Table 5:** Association between murmur intensity and the presence of dimensional changes.

Murmur Intensity	No Dimensional Changes	Dimensional Changes
Total	594 (100%)	228 (100%)
No murmur	147 (25%)	23 (10%)
Murmur Grade 1–2/6	278 (47%)	63 (28%)
Murmur Grade 3–4/6	162 (27%)	117 (51%)
Murmur Grade 5–6/6	7 (1%)	25 (11%)
Median	2.500	3.000
95% Confidence Interval		
Lower Bound	2.474	3.113
Upper Bound	2.635	3.404

**Table 6:** ANOVA for influence factors on murmur intensity; adjusted R-squared = 0.456; data from a study with 735 horses that underwent echocardiography.

Parameter	Regression Coefficient	Standard Error	t	*p*-Value	95% Confidence Interval	
					Lower Bound	Upper Bound
constant	11.060	1.827	6.053	<0.001	7.471	14.650
No MVI	3.619	1.292	2.801	0.005	1.081	6.157
Mild MVI	5.715	1.784	3.203	0.001	2.210	9.220
Moderate MVI	0.462	1.293	0.357	0.721	−2.078	3.001
Severe MVI	0					
No TVI	−8.084	2.165	−3.734	<0.001	−12.337	−3.831
Mild TVI	−9.540	2.337	−4.082	<0.001	−14.132	−4.949
Moderate TVI	−1.767	0.434	−4.072	<0.001	−2.620	−0.915
Severe TVI	0					
No AVI	−1.645	1.382	−1.190	0.235	−4.359	1.070
Mild AVI	−6.915	2.818	−2.454	0.014	−12.449	−1.380
Moderate AVI	0.227	2.054	0.111	0.912	−3.806	4.261
Severe AVI	0					
No VSD	−5.047	1.076	−4.691	<0.001	−7.160	−2.934
VSD	0					
No LA enlargement	−2.638	1.404	−1.878	0.061	−5.397	0.121
Mild LA enlargement	−3.736	1.368	−2.730	0.007	−6.424	−1.048
Moderate LA enlargement	−3.470	1.316	−2.636	0.009	−6.055	−0.884
Severe LA enlargement	0					
No LV enlargement	4.218	1.232	3.423	0.001	1.798	6.639
Mild LV enlargement	4.507	1.244	3.623	<0.001	2.063	6.950
Moderate LV enlargement	2.668	1.196	2.231	0.026	0.319	5.017
Severe LV enlargement	0					
No RA enlargement	0.907	0.446	2.036	0.042	0.032	1.782
Mild RA enlargement	−0.509	0.482	−1.055	0.292	−1.456	0.439
Severe RA enlargement	0					
No RV enlargement	0.495	0.549	0.901	0.368	−0.584	1.574
Mild RV enlargement	−1.505	1.152	−1.306	0.192	−3.768	0.758
Severe RV enlargement	0					
No AVI × No LV enlargement	−4.518	1.121	−4.029	<0.001	−6.721	−2.316
No AVI × Mild LV enlargement	−4.944	1.098	−4.501	<0.001	−7.102	−2.787
No AVI* Moderate LV enlargement	−2.501	1.048	−2.387	0.017	−4.559	−0.443
Mild AVI × No LV enlargement	−0.925	1.492	−0.620	0.535	−3.855	2.005
Mild AVI × Mild LV enlargement	−1.273	1.331	−0.957	0.339	−3.888	1.341
Mild AVI × Moderate LV enlargement	1.163	1.462	0.796	0.427	−1.709	4.035
Moderate AVI × No LV enlargement	−0.185	0.755	−0.245	0.807	−1.669	1.299
Moderate AVI × Moderate LV enlargement	1.432	0.718	1.993	0.047	0.021	2.842
No MVI × No AVI	−2.367	1.081	−2.189	0.029	−4.490	−0.244
No MVI × Mild AVI	−1.080	1.482	−0.728	0.467	−3.991	1.832
No MVI × Moderate AVI	0.354	1.612	0.220	0.826	−2.812	3.520
Mild MVI × No AVI	−1.582	1.216	−1.301	0.194	−3.970	0.806
Mild MVI × Mild AVI	−0.523	0.840	−0.623	0.533	−2.174	1.127
Moderate MVI × No AVI	−0.995	0.972	−1.024	0.306	−2.903	0.914
No TVI × No AVI	6.061	2.213	2.739	0.006	1.714	10.407
No TVI × Mild AVI	6.928	2.479	2.795	0.005	2.059	11.798
No TVI × Moderate AVI	−1.456	1.174	−1.240	0.215	−3.763	0.850
Mild TVI × No AVI	7.759	2.386	3.252	0.001	3.072	12.446
Mild TVI × Mild AVI	8.176	2.651	3.084	0.002	2.968	13.384
No LA enlargement × No RV enlargement	−0.683	0.617	−1.107	0.269	−1.895	0.529
No LA enlargement × Mild RV enlargement	2.573	1.267	2.031	0.043	0.085	5.062
No MVI × No LA enlargement	1.627	0.904	1.799	0.073	−0.149	3.403
No MVI × Mild LA enlargement	3.057	0.737	4.146	<0.001	1.609	4.505
No MVI × Moderate LA enlargement	1.044	0.783	1.334	0.183	−0.494	2.582
Mild MVI × No LA enlargement	−0.718	0.855	−0.839	0.402	−2.398	0.962
Mild MVI × Mild LA enlargement	−0.230	0.666	−0.345	0.730	−1.539	1.079
Mild MVI × Moderate LA enlargement	−1.114	0.732	−1.522	0.128	−2.552	0.323
Moderate MVI × No LA enlargement	−0.217	0.663	−0.328	0.743	−1.520	1.085
Moderate MVI × Moderate LA enlargement	−0.961	0.518	−1.854	0.064	−1.979	0.057
No VSD × No LA enlargement	3.221	1.079	2.984	0.003	1.101	5.341
No VSD × Mild LA enlargement	3.327	1.343	2.477	0.014	0.688	5.966
No VSD × Moderate LA enlargement	4.039	1.252	3.226	0.001	1.579	6.498
No MVI × No LV enlargement	−4.239	1.098	−3.860	<0.001	−6.396	−2.082
No MVI × Mild LV enlargement	−4.109	1.217	−3.375	0.001	−6.500	−1.717
No MVI × Moderate LV enlargement	−3.841	1.105	−3.476	0.001	−6.011	−1.670
Mild MVI × No LV enlargement	−4.382	1.387	−3.159	0.002	−7.106	−1.657
Mild MVI × Mild LV enlargement	−4.629	1.429	−3.239	0.001	−7.437	−1.822
Mild MVI × Moderate LV enlargement	−4.129	1.428	−2.892	0.004	−6.933	−1.324
Moderate MVI × No LV enlargement	0.522	0.705	0.740	0.460	−0.863	1.906
Moderate MVI × Mild LV enlargement	0.704	0.787	0.894	0.372	−0.843	2.250

Abbreviations: MVI = Mitral valve regurgitation, AVI = Aortic valve regurgitation, TVI = Tricuspid valve regurgitation, PVI = Pulmonal valve regurgitation, LA = Left Atrium, LV = Left Ventricle, RA = Right Atrium, RV = Right Ventricle.

## Data Availability

The data is stored in the archives of the equine clinic in Bargteheide, as we retrospectivel analysed it.

## Abbreviations

MVI	Mitral Valve Regurgitation
AVI	Aortic Valve Regurgitation
TVI	Tricuspid Valve Regurgitation
PVI	Pulmonal Valve Regurgitation
VSD	Ventricular Septal Defect
LA	Left Atrium
LV	Left Ventricle
RA	Right Atrium
RV	Right Ventricle
OR	Odds Ratio
mm	Millimeters
